# Prognostic utility of copy number variations in circulating tumor DNA in head and neck cancer

**DOI:** 10.1016/j.jlb.2026.100668

**Published:** 2026-10-01

**Authors:** Edward S. Sim, Daniel A. Ruiz-Torres, Pan Du, Yong Huang, Frank Zhang, Shidong Jia, Adam S. Fisch, Ross D. Merkin, Jonathan J. Paly, Daniel L. Faden

**Affiliations:** a Department of Otolaryngology-Head and Neck Surgery, Massachusetts Eye and Ear, Boston, MA, USA; b Department of Otolaryngology-Head and Neck Surgery, Harvard Medical School, Boston, MA, USA; c Broad Institute of MIT and Harvard, Cambridge, MA, USA; d Predicine, Hayward, CA, USA; e Departments of Pathology, Massachusetts General Hospital and Harvard Medical School, Boston, MA, USA; f Department of Medicine, Harvard Medical School, Boston, MA, USA; g Center for Head and Neck Cancers, Massachusetts General Hospital, Boston, USA; h Department of Radiation Oncology, Massachusetts General Hospital, Boston, USA

**Keywords:** Head and neck squamous cell carcinoma, Circulating tumor DNA, Minimal residual disease, Liquid biopsy, Next-generation sequencing

## Abstract

**Background:**

Plasma circulating tumor DNA (ctDNA) is a minimally invasive biomarker in head and neck squamous cell carcinoma (HNSCC). Most commercially available assays for human papillomavirus-unrelated HNSCC are tumor-informed assays that require tissue sampling and may not comprehensively capture tumor heterogeneity. We evaluated a tumor-agnostic approach using genome-wide ctDNA copy-number variation (CNV) profiling to derive blood copy-number burden (CNB) for prognostication in non-surgically treated HNSCC.

**Methods:**

ctDNA was extracted from 250 longitudinal plasma samples from 33 patients and analyzed using PredicineSCORE low-pass whole-genome sequencing (LP-WGS). CNB was dichotomized as positive (CNB+) or negative (CNB−) using a predefined threshold. Associations with overall survival (OS) and progression-free survival (PFS) were assessed with Kaplan–Meier models.

**Results:**

Median age was 63 years; 67% were male; 67% had recurrent/metastatic disease; 15% were HPV-associated. Treatment included chemoradiation (39%) and immune checkpoint blockade–based regimens (60%). The most frequent CNV was 3q gain. Baseline plasma was available for 30 patients, of whom 17 (56%) were CNB+. Baseline CNB+ was associated with inferior OS (HR 4.38, 95% CI 1.22–15.72; p = 0.01) but not PFS (HR 1.41, 95% CI 0.62–3.20; p = 0.41) over a median follow-up of 23.4 months. CNB + at any post-treatment timepoint occurred in 26/33 (79%) and showed a non-significant trend toward worse OS (HR 3.03; p = 0.12) and PFS (HR 2.70; p = 0.06). Detection rates were modest, highlighting potential utility mainly in CNV-high, HPV-independent tumors over time.

**Conclusions:**

LP-WGS enables tumor-agnostic detection of CNV signals in plasma from non-surgically treated HNSCC, and baseline CNB stratifies OS. Larger, more homogeneous cohorts with matched tumor sequencing are needed to validate ctDNA-derived CNV metrics and define which molecular subtypes benefit most from blood-based CNB detection and monitoring.

**Level of evidence:**

3.

## 1 Introduction

Plasma circulating tumor DNA (ctDNA) has emerged as a promising, minimally invasive biomarker for predicting outcomes and monitoring treatment efficacy across solid cancers including head and neck squamous cell carcinoma (HNSCC). However, tumor-informed ctDNA assays require sequencing of the primary tumor, which may not fully reflect the heterogeneity or subclonal architecture of the tumor due to sampling bias. Further, tumor-informed approaches are subject to tissue accessibility constraints, especially in the setting of HNSCC patients treated with non-surgical modalities in which surgical tissue specimens may not be accessible. In contrast, unbiased, genome-wide ctDNA profiling offers a tumor-agnostic strategy that may provide a more comprehensive approach to prognostication and disease monitoring. A tumor's copy number variation (CNV) profile is known to carry prognostic potential and can be detected in a tumor-agnostic fashion [[1], [2], [3], [4]]. Herein, we aim to assess the utility of genome-wide, next-generation sequencing of plasma ctDNA to quantify CNV signals as a potential tumor-agnostic approach for prognostic assessment in non-surgically treated HNSCC patients.

In this study, ctDNA was isolated from 250 plasma samples collected longitudinally from 33 HNSCC patients (Fig. 1A). PredicineSCORE, a low-pass whole-genome sequencing (LP-WGS) assay at 3-5x coverage, was performed to detect CNVs in plasma samples at baseline and at serial timepoints following treatment initiation [[5], [6], [7], [8], [9], [10], [11], [12], [13], [14]]. We used ichorCNA to estimate CNVs in ctDNA derived from patient plasma from GC- and mappability-normalized reads, applying a hidden Markov model [15]. For each 1 Mb segment, copy number deviation was calculated as the log2 ratio of normalized reads in the test sample versus a pool of normal plasma samples. Each sample's copy number burden (CNB) score was then defined as the logarithm of the sum of absolute CNV z-scores, with higher scores indicating greater CNV abnormality relative to normal background [16]. A CNB cutoff was defined as three standard deviations above the mean CNB of plasma samples derived from patients without cancer, and samples above or below this threshold were considered CNB positive (CNB+) or negative (CNB-) respectively [16]. Kaplan-Meier methods and Cox proportional hazards modeling were used to assess the association of CNB status with overall survival (OS) and progression-free survival (PFS).

**Fig. 1:**
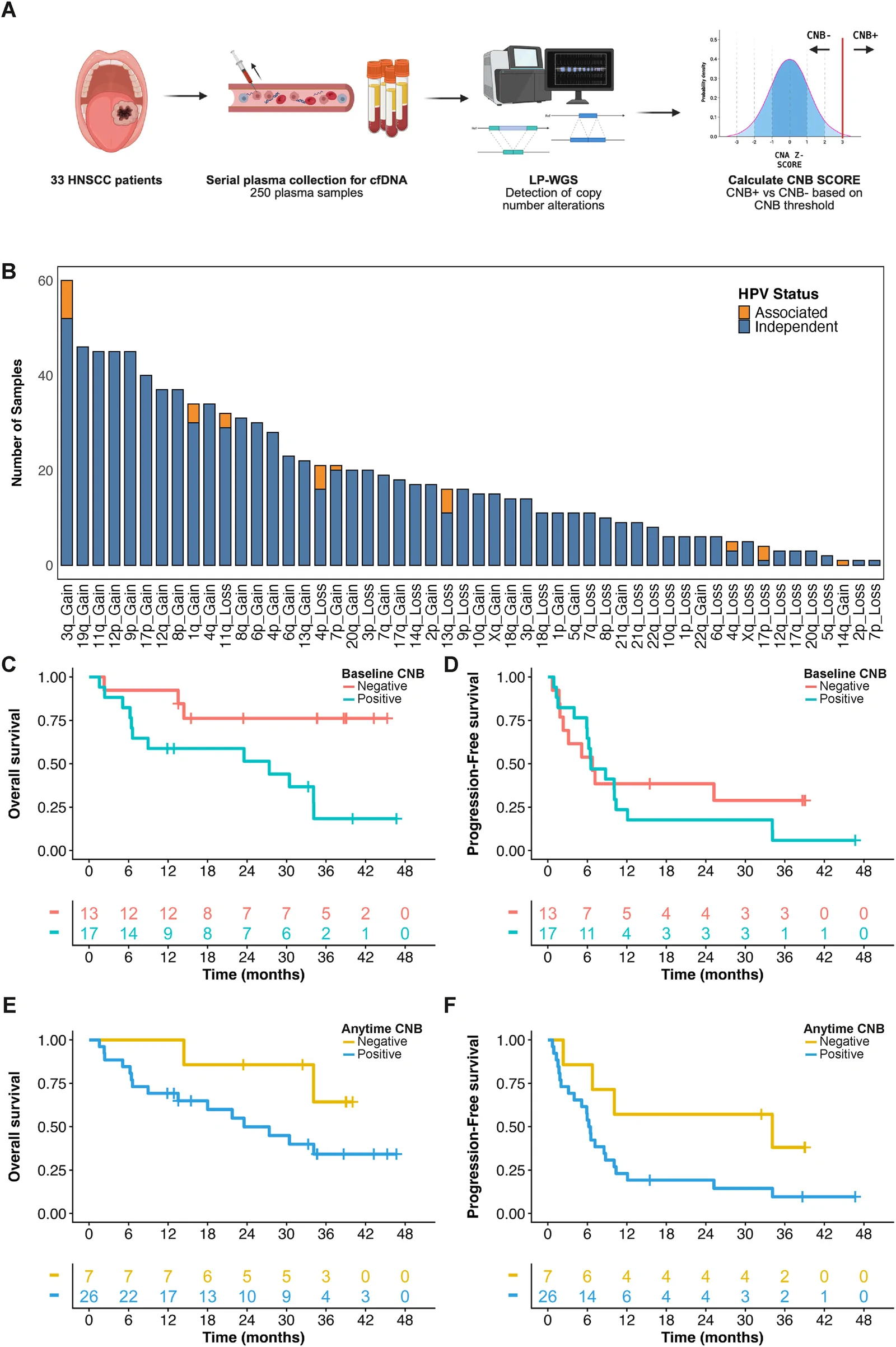
A) Schematic of study design. Plasma from patients with HNSCC was sampled at baseline and at serial timepoints following treatment initiation and analyzed by low-pass whole-genome sequencing (LP-WGS) to detect copy number alterations B) Landscape of copy number alterations across samples. Orange bars indicate samples from patients with HPV-associated HNSCC while blue bars indicate samples from patients with HPV-independent tumors. C/D) Kaplan-Meier curves for overall (C) and progression-free survival (D) stratified by baseline CNB status (CNB+ in blue, CNB- in red) at baseline before the start of treatment. E/F) Kaplan-Meier curves for overall (E) and progression-free (F) survival stratified by patients who were CNB+ (blue) or CNB- (yellow) at any timepoint after the start of treatment.

## 2 Discussion

The median age at treatment initiation was 63 years (range, 34–91). Most patients were male (22/33, 67%) and non-Hispanic White (28/33, 85%). Eighteen patients (54%) were current or former smokers. Five patients (15%) had human papillomavirus (HPV)-associated disease. Most patients had recurrent and/or metastatic disease (22/33, 67%). Of the eleven patients (33%) with primary disease, one (1/11, 9%) had American Joint Committee on Cancer (AJCC) 8th edition clinical stage II disease, two (2/11, 18%) had stage III disease, and 8 (8/11, 73%) had stage IVa–IVb disease. The oral cavity was the most common anatomic subsite (16/33, 48%), Thirteen patients (39%) received chemoradiation, twelve (36%) immune checkpoint blockade (ICB) monotherapy, six (18%) ICB plus chemotherapy, and two (6%) ICB plus a novel agent in a clinical trial (Table 1). The most frequent chromosomal CNV was a gain of 3q, observed in 8 of 48 HPV-associated plasma samples (17%) and in 52 of 202 HPV-independent) samples (26%) [Fig. 1B].

**Table 1:** Patient clinical and disease characteristics.

Characteristic	Total (n = 33)
*Median Age – years (range)*	63 (34–91)
*Male sex – no. (%)*	22 (67)
*Race – no (%)*	
White	28 (85)
Asian	1 (3)
Declined	4 (12)
*Tobacco – no. (%)*	
Current	2 (6.1)
Former	16 (48)
Never	15 (45)
*Tumor stage – no. (%)*	
Recurrent/Metastatic	22 (67)
Primary	11 (33)
*Primary site – no. (%)*	
Oral Cavity	16 (48)
Oropharynx/Pharynx	8 (24)
Sinonasal	5 (15)
Larynx/Hypopharynx	2 (6)
Unknown Primary	2 (6)
*HPV Status – no. (%)*	
Independent	28 (85)
Associated	5 (15)
*Treatment – no. (%)*	
CRT	13 (39)
ICB monotherapy	12 (36)
ICB + Chemotherapy	6 (18)
ICB + novel agent	2 (6)

Thirty patients had baseline plasma available with 17 (56%) meeting the CNB threshold. Fourteen of these patients (14/17, 83%) were HPV-independent and eleven (11/17, 65%) had recurrent/metastatic disease. The median follow-up for the entire cohort was 23.4 months (IQR: 11.9-34.6 months). Baseline CNB positivity was associated with significantly worse OS (HR = 4.38; 95% CI = 1.22–15.72; p = 0.01; 10 deaths). Multivariate analysis controlling for tumor stage and HPV status demonstrated that this association remained significant (adjusted HR = 5.59; 95% CI 1.49–20.99; p = 0.046). No significant difference was detected in PFS (HR = 1.41; 95% CI = 0.62-3.20; p = 0.41; 25 events) [Fig. 1C and D]. CNB positivity detected at any point after treatment was observed in 26/33 patients (79%) and showed a non-significant trend towards worse OS (HR = 3.03; 95% CI = 0.69-13.33; p = 0.12; 17 deaths) and PFS (HR = 2.70; 95% CI = 0.92-7.89; p = 0.06; 27 events) [Fig. 1E and F].

## 3 Conclusion

Using a LP-WGS assay, we demonstrate that CNVs can be detected in ctDNA of HNSCC patients receiving non-surgical treatment, although at limited rates. The sensitivity of CNV detection using LP-WGS is highly influenced by the fraction of ctDNA in the total cell-free DNA (cfDNA) compartment of plasma and sequencing depth, particularly for lower-amplitude or focal alterations, limiting utility in low tumor fractions samples. Additionally, LP-WGS sequencing of cfDNA without a paired control on white blood cell DNA cannot reliably distinguish tumor-derived copy number alterations from signals derived from clonal hematopoiesis.

Despite these limitations, CNB positivity at baseline was independently associated with OS (adjusted HR = 5.59; 95% CI 1.49–20.99; p = 0.046) after controlling for HPV status and tumor stage, although this finding will require validation in larger, prospective studies with more homogeneous cohorts. Interestingly, while the association with OS was significant, no significant association was observed for PFS, likely reflecting limited power to detect PFS differences. Furthermore, this study's limited sample size and few number of events also likely contributed to the lack of statically significant difference in survival between CNB+ and CNB- groups at post-treatment timepoints despite the separation in Kaplan-Meier curves. Additional limitations included the heterogeneity in clinical characteristics and treatment modalities within the cohort, incomplete availability of baseline samples, and the absence of matched tumor sequencing data, limiting our ability to directly assess tumor CNV status to validate ctDNA-derived CNV profiles.

Comprehensive genomic profiling in HNSCC has consistently shown frequent CNVs, particularly in 3q, which includes *PIK3CA*, *SOX2* and *TP63*, three of most altered genes in HNSCC [17]. In our data, we similarly observed that the most common alterations in patients’ plasma involved chromosomes 3q. HPV-independent HNSCC has been shown to be composed of distinct molecular subtypes characterized by high (CNV-high) or low (CNV-silent) levels of CNVs, while CNVs are less frequent in HPV-associated HNSCC [17]. CNV-high subtypes are associated with poorer prognosis as they are often more poorly-differentiated and have a more immunosuppressed tumor microenvironment compared to CNV-silent subtypes [18]. Concordantly, patients in our cohort with high CNB in plasma exhibited worse survival. As HNSCC have distinct subtypes based on CNV profiles, CNV-based ctDNA monitoring may be most informative in CNV-high HPV- independent tumors, whereas integrative approaches with additional ctDNA methods such as fragmentomics, methylation, and multidimension approaches may be beneficial for prognostication CNV-silent or HPV-associated subtypes [19,20].

## Ethics declaration

Written informed consent to take part in the study and to publish the article has been obtained from all participants or their legal representatives. The privacy rights of participants have been observed.

This study included organ or tissue donors. This study includes human biological material and consent was obtained by donors, or their next of kin or legal representatives, for use in this study and for publication of the article. The samples used in this research were not sourced from executed prisoners or prisoners of conscience.

This study was performed in compliance with relevant laws, regulatory frameworks and guidelines where the research took place. This study was approved by the IRB. (Approval No. DFHCC 18653)

## Funding/disclosures:

**E.S. Sim** reports funding support from the NIDCD T32DC000020. **A.S. Fisch** reports grant support from Haystack Oncology, Inc. outside the submitted work. **D.L. Faden** reports grants from the NIH/NIDCR K23 DE029811, NIH/NIDCR R03 DE030550, and NIH/NCI R21 CA267152 during the conduct of the study as well as grants from Bristol Myers Squibb, Calico, and Haystack (Quest); other support from Predicine, BostonGene, and Neogenomics; and personal fees from Merck, Chrysalis Biomedical Advisors, Natera and Arcadia outside the submitted work; in addition, **D.L. Faden** has a patent for 169511-00058-MEEI2024-32 pending. No other disclosures were reported.

## Declaration of competing interests

**E.S. Sim** reports funding support from the NIDCD T32DC000020. **A.S. Fisch** reports grant support from Haystack Oncology, Inc. outside the submitted work. **D.L. Faden** reports grants from the NIH/NIDCR K23 DE029811, NIH/NIDCR R03 DE030550, and NIH/NCI R21 CA267152 during the conduct of the study as well as grants from Bristol Myers Squibb, Calico, and Haystack (Quest); other support from Predicine, BostonGene, and Neogenomics; and personal fees from Merck, Chrysalis Biomedical Advisors, Natera and Arcadia outside the submitted work; in addition, **D.L. Faden** has a patent for 169511-00058-MEEI2024-32 pending. No other disclosures were reported.
